# Comparative Metabolomic Studies of Siberian Wildrye (*Elymus sibiricus* L.): A New Look at the Mechanism of Plant Drought Resistance

**DOI:** 10.3390/ijms24010452

**Published:** 2022-12-27

**Authors:** Qingqing Yu, Yi Xiong, Xiaoli Su, Yanli Xiong, Zhixiao Dong, Junming Zhao, Xin Shu, Shiqie Bai, Xiong Lei, Lijun Yan, Xiao Ma

**Affiliations:** 1 College of Grassland Science and Technology, Sichuan Agricultural University, Chengdu 611130, China; 2 Sichuan Academy of Grassland Science, Chengdu 610097, China

**Keywords:** metabolomics, *Elymus sibiricus*, drought stress, proline, flavonoids

## Abstract

Drought is one of the most important factors affecting plant growth and production due to ongoing global climate change. *Elymus sibiricus* has been widely applied for ecological restoration and reseeding of degraded grassland in the Qinghai–Tibetan Plateau (QTP) because of its strong adaptability to barren, salted, and drought soils. To explore the mechanism of drought resistance in *E. sibiricus*, drought-tolerant and drought-sensitive genotypes of *E. sibiricus* were used in metabolomic studies under simulated long-term and short-term drought stress. A total of 1091 metabolites were detected, among which, 27 DMs were considered to be the key metabolites for drought resistance of *E. sibiricus* in weighted gene co-expression network analysis (WGCNA). Ten metabolites, including 3-amino-2-methylpropanoic acid, coniferin, R-aminobutyrate, and so on, and 12 metabolites, including L-Proline, L-histidine, N-acetylglycine, and so on, showed differential accumulation patterns under short-term and long-term drought stress, respectively, and thus, could be used as biomarkers for drought-tolerant and drought-sensitive *E. sibiricus*. In addition, different metabolic accumulation patterns and different drought response mechanisms were also found in drought-tolerant and drought-sensitive genotypes of *E. sibiricus*. Finally, we constructed metabolic pathways and metabolic patterns for the two genotypes. This metabolomic study on the drought stress response of *E. sibiricus* can provide resources and a reference for the breeding of new drought-tolerant cultivars of *E. sibiricus*.

## 1. Introduction

*Elymus* L. sensu lato is the largest, most morphologically diverse, and most widely distributed genus in the Triticeae, including approximately 150 perennial, allopolyploid species occurring worldwide in non-tropical areas [1]. *Elymus sibiricus* (Siberian wildrye), the model species of the genus *Elymus*, is a perennial, cool-season, and self-pollinated forage grass with the genome constitution of StStHH (2n = 4x = 28) [2]. Given the characteristics of high biomass, good forage quality, and excellent adaptation to various environmental situations of high altitude [3], the *E. sibiricus* were often used in artificial grassland construction and revegetation of degraded grassland in Qinghai–Tibet Plateau (QTP) areas as the main grass species [4,5]. Most importantly, *E. sibiricus* occurs naturally in various habitats at altitudes of 1500–4900 m, especially in drought-prone areas such as Xinjiang, Inner Mongolia, and the QTP, which become more and more arid with the changes of the global climate. For example, 63% of the agricultural and pastoral areas in the QTP region are drought-prone areas with medium or higher risk levels [6]. The great adaptation to those drought areas enables *E. sibiricus,* the excellent research object, to excavate the drought-tolerance mechanisms. However, given the lack of reference genomic information for *E. sibiricus*, the research on the drought tolerance and mechanisms of *E. sibiricus* were underdeveloped, and only a few studies based on de novo transcriptome profiles identified some drought-response genes [7].

Drought resistance of plants involves many physiological and molecular processes, and a series of responses such as gene expression and cellular metabolism [8,9]. At present, metabolomics (also known as metabolic phenotyping or metabolic profiling) has become an established technique in academic research regarding abiotic stress of plants. It can provide complementary information for genomics, transcriptomics, and proteomics research, and thus, can act as an alternative strategy for stress-related studies that can be effectively performed on any species whether they have a reference genome or not [10,11,12]. Therefore, metabolomics plays an increasingly important role in plant biotic and abiotic stress [13], especially in the discovery of biomarkers and in plant phenotype analysis [14]. Especially, many technical approaches, such as gas chromatography (GC), liquid chromatography (LC), mass spectrometry (MS), and ultra−performance liquid chromatography−tandem mass spectrometry (UPLC–MS/MS), have already been successfully applied to the isolation and identification of metabolites. In past metabolomics studies, only quantitative (targeted) metabolism and stoichiometric (untargeted) metabolomics routes were used for metabolomics experiments [12]. In recent years, with the continuous development of technology, an experimental method called widely targeted metabolomics has emerged [15,16,17], which integrates the advantages of high sensitivity and precise quantification of targeted metabolic technology and high resolution and wide coverage of untargeted metabolic technology. The widely targeted metabolism has the advantages of more identified substances, accurate qualitative and quantitative, high sensitivity, and good repeatability. It provides a more efficient method for the detection of complex metabolites in animals and plants.

In this study, a widely targeted metabolome was used to study the response to drought stress in two *E. sibiricus* genotypes. The purpose of this study was to (i) screen the drought-tolerant genotype of *E. sibiricus* to provide germplasm resources for the breeding of drought-tolerant varieties; (ii) identify drought-responsive metabolites and key drought-tolerant metabolites of *E. sibiricus*; (iii) compare the metabolic responses of two genotypes of *E. sibiricus* under long-term and short-term drought pressures; (iv) metabolic patterns were compared between two different genotypes and the key metabolic pathway was found to response drought-tolerant in *E. sibiricus*. A metabolic model and pathway of *E. sibiricus* in response to drought pressure is proposed.

## 2. Results

### 2.1. Screening of Drought-Tolerant and Sensitive Genotypes of Elymus sibiricus

A total of 37 *E. sibiricus* accessions were treated with 20% PEG-6000 to evaluate their drought tolerance, among which, XJ030-21 and W16-29 possessed the highest and lowest values in membership function analysis (Table S1). In addition, these two genotypes exhibited great phenotypic differences, with phenotype scores of 4.65 in XJ030-21 and 1.33 in W16-29. Furthermore, XJ030-21 had the highest RWC (89.35%) and a lower REC (19.21%), and W16-29 had the lowest RWC (14.22%) and a higher REC (89.10%) (Figure S1). Therefore, these two genotypes were considered as the drought-tolerant (X) and drought-sensitive (W) genotypes for further study.

**Figure 1:**
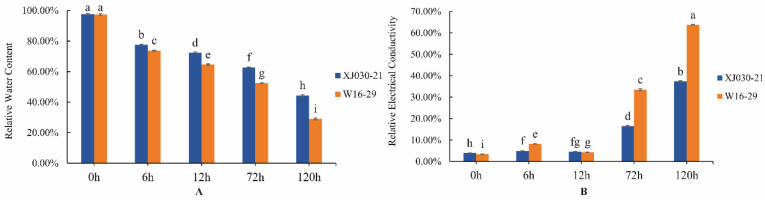
Related physiological indicators of drought-tolerant (X) and drought-sensitive (W) genotypes. (**A**): Relative water content (RWC). (**B**): Relative electrical conductivity (REC). Different lower-case letters in the figure indicate significant difference at level of *p* < 0.05.

The RWC values of the drought-tolerant genotype and drought-sensitive genotype significantly decreased continuously with the extension of drought stress (*p* < 0.05) and the RWC values of the drought-tolerant genotype were significantly higher than those of the drought-sensitive genotype under drought stress (Figure 1A). Under short-term (6 and 12 h) drought stress conditions, the REC of the two genotypes showed a slight increase, which was significantly different from the CK. Under the long-term (72 and 120 h) stress treatment, the REC of the two genotypes increased greatly, and the increase of the drought-sensitive genotype (W) was much greater than that of the drought-tolerant genotype (X) (Figure 1B). In conclusion, it is reliable to select XJ030-21 and W16-29 as the drought-tolerant and drought-sensitive genotypes of *E. sibiricus*, respectively, for subsequent metabolomics studies.

### 2.2. Metabolite Detection and Quality Control

**Figure 2:**
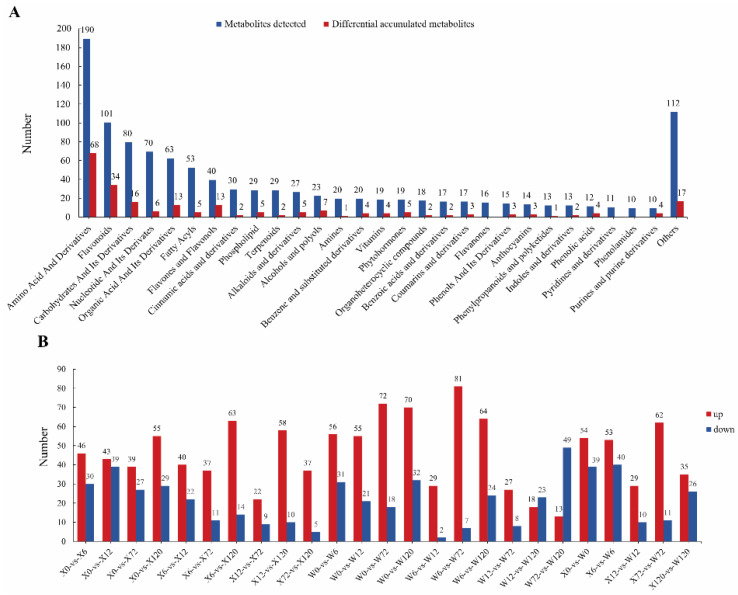
Number of metabolites histogram, (**A**): All metabolites are classified in blue and their amounts, red is the number and classification of DMs; (**B**): differential metabolites of 25 comparison groups, up-regulated in red and down-regulated in blue.

In order to study the metabolic response of *E. sibiricus* under drought stress, leaf samples of two genotypes of *E. sibiricus* treated with PEG-6000 for 0, 6, 12, 72, and 120 h were used to detect their metabolite levels. A total of 1091 metabolites were detected in all 30 samples under drought stress (Table S2), and all these metabolites could be further divided into 29 classes, which included 190 amino acids and their derivatives, 101 flavonoids, 80 carbohydrates and their derivatives, 70 nucleotides and their derivates, 63 organic acids and their derivatives, 53 fatty acyls, 40 flavones and flavonols, 30 cinnamic acids and their derivatives, 29 phospholipids, 29 terpenoids, 27 alkaloids and their derivatives, 23 alcohols and polyols, 20 amines, 20 benzenes and their substituted derivatives, 19 vitamins, 19 phytohormones, 18 organoheterocyclic compounds, 17 benzoic acids and their derivatives, 17 coumarins and their derivatives, 16 flavanones, 15 phenols and their derivatives, 14 anthocyanins, 13 phenylpropanoids and polyketides, 13 indoles and their derivatives, 12 phenolic acids, 11 pyridines and their derivatives, 10 phenolamides, 10 purines and purine derivatives and 112 other metabolites (Figure 2A). A total of 396 metabolites were annotated using the Kyoto Encyclopedia of Genes and Genomes (KEGG) compound database.

**Figure 3:**
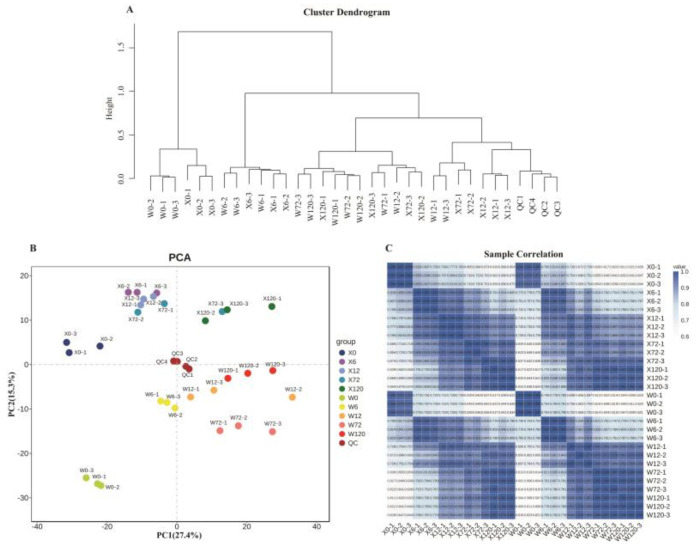
Sample quality control diagram, (**A**): Hierarchical clustering of 30 samples; (**B**): 2D map of principal component analysis; (**C**): Pearson’s correlation heatmap of 30 samples.

For further analysis of 1091 metabolites detected in thirty samples, we performed hierarchical cluster analysis and principal component analysis (PCA) with four quality control (QC) samples and thirty test samples, and the results showed that X0 and W0 were just separated from the other samples (Figure 3A,B). Furthermore, the first and second principal components of the principal component analysis explained 42.7% of the total variance, thus clearly distinguishing the two genotypes of *E. sibiricus* (Figure 3B), which indicated that the accumulation of metabolites detected in the two genotypes of *E. sibiricus* changed under drought stress. Both analyses showed small differences within each treatment, but large differences among the different treatments. A heatmap based on Pearson’s correlation coefficient among thirty samples was constructed (Figure 3C), which showed a highly significant positive correlation among the three tested biological replicates. These results suggested that our method correctly revealed the metabolic basis of *E. sibiricus* under drought stress, and the experimental design and data are very reliable for downstream analysis.

### 2.3. Identification of Differential Metabolites in Multiple Comparison Groups

To identify differential metabolites (DMs), orthogonal partial least squares discriminant analysis (OPLS−DA) was performed on the 25 comparison groups. The metabolites with VIP ≥ 1 were determined as DMs, and the differential metabolites when Log_2_(FC) was greater than 0 and less than 0 were up-regulated and down-regulated, respectively. A total of 231 DMs were identified in 25 tested comparison groups (Figure 2B, Table S3), which showed different accumulation patterns under drought stress. It is worth noting that amino acids and their derivatives (68/190, 35.79%), flavonoids (34/101, 33.66%), flavones and flavonols (13/40, 32.5%), alcohols and polyols (7/23, 30.43%), phenolic acids (4/12, 33.33%) and purines and purine derivatives (4/10, 40%) were significantly affected by drought stress (Figure 2B). Interestingly, flavanones (0%, 0/16), pyridines and their derivatives (0%, 0/11) and phenol amides (0%, 0/10) did not accumulate significantly under drought stress (Figure 2B), which indicated that the accumulation of these three classes is not induced by drought stress in *E. sibiricus*.

**Figure 4:**
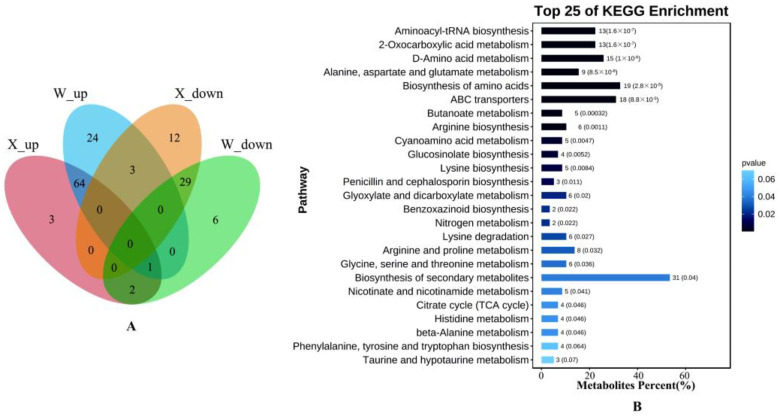
(**A**) Venn diagrams of differential metabolites up-regulated by drought-tolerant genotype (X) and drought-sensitive genotype (W); (**B**): KEGG enrichment pathway map of DMs in 8 comparison groups.

For intuitive understanding of the number of DMs in the eight comparison groups (X0 vs. X6, X0 vs. X12, X0 vs. X72, X0 vs. X120, W0 vs. W6, W0 vs. W12, W0 vs. W72, and W0 vs. W120), Venn diagrams of DMs based on the up-regulation and down-regulation of the drought-tolerant genotype (X) and the up-regulation and down-regulation of the drought-sensitive genotype (W) were constructed (Figure 4A). After removing the duplicated metabolites, 144 differential metabolites were identified in these eight comparison groups. KEGG pathway enrichment analysis of the 144 DMs showed that DMs drought-stressed DMs exhibited significant enrichment across 23 pathways, including aminoacyl-tRNA biosynthesis, 2-oxocarboxylic acid metabolism, D-amino acid metabolism, alanine, aspartate and glutamate metabolism, biosynthesis of amino acids, ABC transporters, butanoate metabolism, arginine biosynthesis, cyanoamino acid metabolism, glucosinolate biosynthesis, and so on (Figure 4B). Furthermore, 114 and 130 DMs were present in the drought-tolerant genotype (X) and drought-sensitive genotype (W), respectively, and 99 common DMs were detected in the drought-tolerant genotype (X) and drought-sensitive genotype (W), in which, 64 common DMs were co-upregulated in the drought-tolerant genotype (X) and drought-sensitive genotype (W), and 29 were co-downregulated. This suggests that these 93 metabolites might be important candidate metabolites for the response to drought in *E. sibiricus*.

In addition, we analyzed the differential metabolites of the two genotypes (X vs. W) and found that there were 47 and 17 DMs down-regulated and up-regulated (Table S4), respectively. Among these, there were 3.96% (4/101) and 17.82% (18/101) flavonoids down-regulated and up-regulated (Table S4), respectively, which indicated that there was up-regulated or no difference in most of flavonoids in drought-sensitive (W) genotype when compared with the drought-tolerant (X) genotype.

### 2.4. Key Metabolites of E. sibiricus in Response to Drought Stress

**Figure 5:**
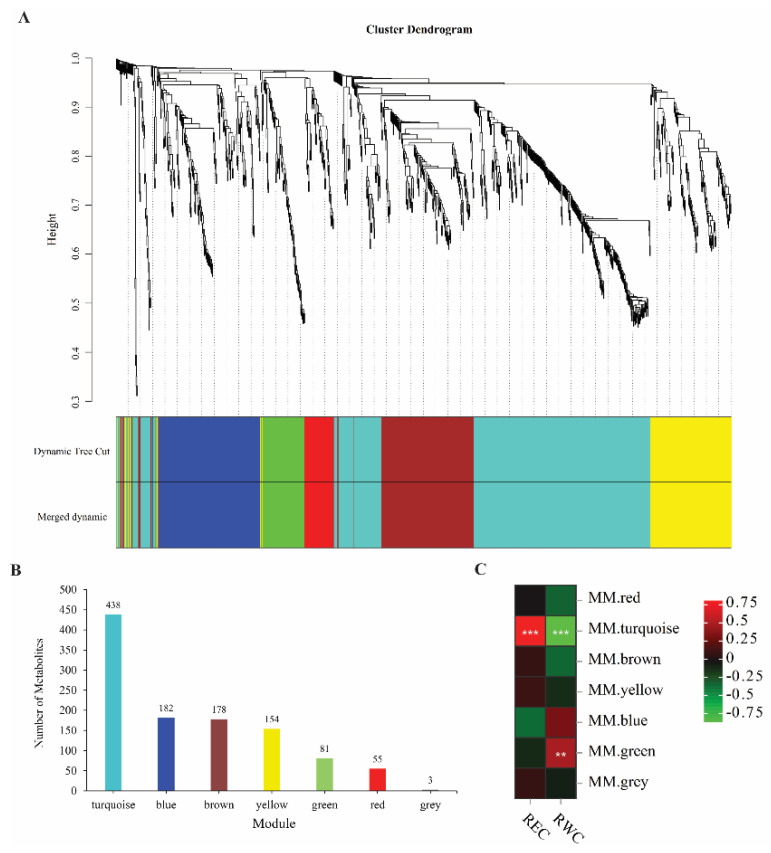
Graph of WGCNA results based on 1091 metabolites with REC and RWC, (**A**): Module Diagram of WGCNA, 7 different colors represent 7 different modules; (**B**): Histogram of the number of metabolites in each module; (**C**): Pearson’s correlation heatmap of 7 modules with REC and RWC, red is positive correlation, green is negative correlation, the darker the color, the stronger the correlation (*** means *p* < 0.001; ** means *p* < 0.01).

**Figure 6:**
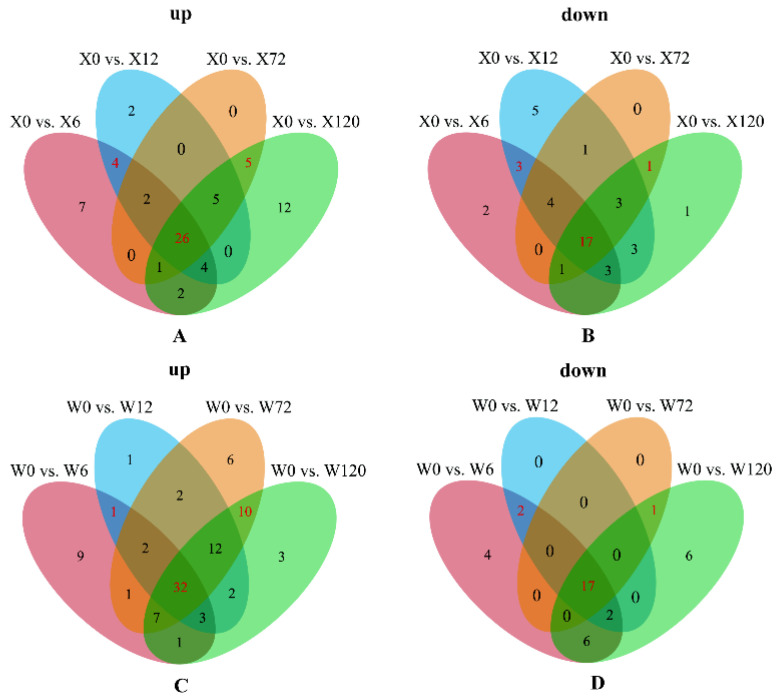
Venn diagram showing the number of up-regulated (**A**) for drought-tolerant genotype (X) and (**C**) for drought-sensitive genotype (W) and down- regulated (**B**) for drought-tolerant genotype (X) and (**D**) for drought-sensitive genotype (W) DMs between drought-tolerant genotype (X) and drought-sensitive genotype (W).

All 1091 metabolites were used for WGCNA to explore the key metabolites of *E. sibiricus* under drought stress. The results showed that the 1091 metabolites could be divided into seven modules (Figure 5A), with 456 metabolites belonging to the turquoise module, 182 metabolites belonging to the blue module, 178 metabolites belonging to the brown module, 154 metabolites belonging to the yellow module, 81 metabolites belonging to the green module, 55 metabolites belonging to the red module, and 3 metabolites belonging to the gray module (Figure 5B). Further analysis based on Pearson’s correlation among modules with REC and RWC found that the turquoise module was extremely significantly correlated with REC and RWC, while only RWC was significantly correlated with the green module (Figure 5C). Therefore, we considered the metabolites with correlation coefficients greater than 0.9 and P values less than 0.001 in these two modules as key candidate metabolites in the response to drought of *E. sibiricus*. Finally, 67 and 18 candidate metabolites were identified in the turquoise and green modules, respectively (Table S5). Moreover, Venn diagrams of differential metabolites for the four treatments compared to controls of drought-tolerant genotype (X) and drought-sensitive genotype (W) were constructed, respectively, to show common DMs (Figure 6). The results showed that there were 26 and 17 metabolites co-upregulated and downregulated among the four comparison groups in the drought-tolerant genotype (X), respectively, while 32 were co-upregulated and 17 were co-downregulated in the drought-sensitive genotype (W), respectively. These results indicate that drought-sensitive genotypes need to upregulate more metabolites in response to drought stress. Further analysis of common DMs in the drought-tolerant genotype (X) and drought-sensitive genotype (W) revealed 22 up-regulated and 13 down-regulated metabolites in both the drought-tolerant genotype (X) and drought-sensitive genotype (W). Combining the results of 85 candidate metabolites in the WGCNA, 16 up-regulated and 11 down-regulated key metabolites were identified in response to drought in *E. sibiricus* (Table S6).

### 2.5. Metabolic Patterns of Two E. sibiricus Genotypes

In order to explore the metabolic patterns of the two *E. sibiricus* genotypes under different drought-induced periods, Venn diagrams of DMs were constructed to understand the metabolic differences more intuitively between the two genotypes under long-term and short-term drought stress (Figure 6). For drought resistant genotypes (X), 26 common up-regulated and 17 common down-regulated metabolites were detected under long-term and short-term drought stress (Figure 6A,B), indicating that the accumulation of these 43 metabolites (Table S7) in the drought-tolerant genotype (X) was mainly induced by drought. Similarly, 49 metabolites (Table S8) in the drought-sensitive genotype (W) were mainly induced by drought. In addition, 3-amino-2-methylpropanoic acid, coniferin, R-aminobutyrate, galactinol, Dl-glutamic acid, vitexin and trigonelline showed differential accumulation in the drought-tolerant genotype (X) under short-term drought, while L-glutamic acid, L-arginine and L-ornithine showed differential accumulation in the drought-sensitive genotype (W) under short-term drought. We suggest that these DMs (not only cumulative quantities, but also types) can be used as biomarkers for drought-tolerant and drought-sensitive *E. sibiricus* under short-term drought stress. Similarly, L-proline, L-histidine, N-acetylglycine and betaine in drought-tolerant genotype (X) and N2-acetyl-L-lysine, 3-ureidopropionate, vitamin U, 1,2-benzenedicarboxylic acid, IAA-Asp, 1-methylpiperidine-2-carboxylic acid, L-proline, and D-proline betaine in drought-sensitive genotype (W) can be used as biomarkers for drought-tolerant and drought-sensitive *E. sibiricus* under long-term drought stress. Interestingly, succinic anhydride induced down-regulation by long-term drought in both the drought-tolerant genotype (X) and drought-sensitive genotype (W), we propose to judge whether the *E. sibiricus* is under long-term drought stress by detecting the down-regulation of succinic anhydride.

**Figure 7:**
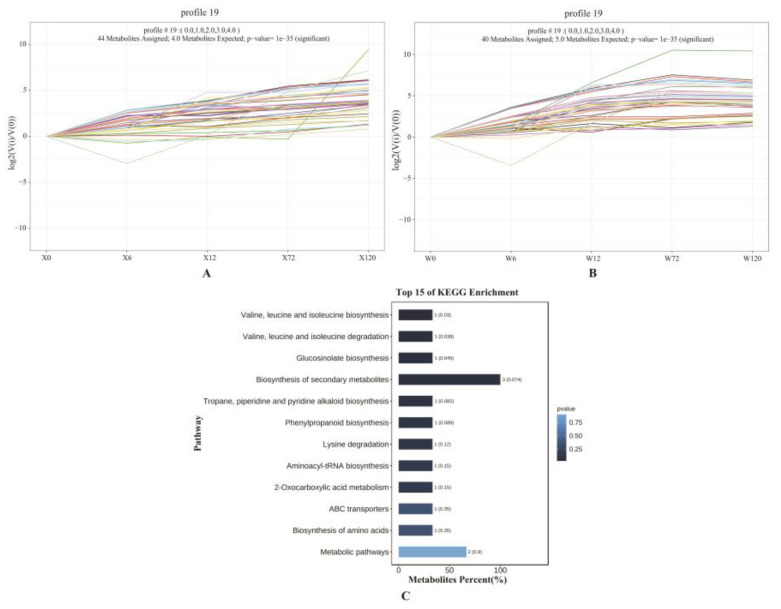
Trend analysis of DMs in drought-tolerant genotype (**A**) and drought-sensitive genotype (**B**); (**C**): KEGG enrichment of DMs in profile 19, which was specific to drought-tolerant genotype.

A total of 114 and 130 DMs in the drought-tolerant genotype (X) and drought-sensitive genotype (W) (Figure 4A) were used for trend analysis, respectively, to explore the metabolic accumulation patterns and differences of different genotypes of *E. sibiricus* under drought stress. An identical profile (profile 19) was found in X and W (Figure 7A,B), and 44 and 40 DMs were found in the drought-tolerant genotype (X) and drought-sensitive genotype (W), respectively. Eight DMs were found to be present only in the drought-tolerant genotype (X) by comparing and removing duplicates, among which there were two forms of leucine (DL-leucine, L-leucine). In order to further explore the drought-tolerant metabolic mechanism of *E. sibiricus*, KEGG enrichment analysis was performed with only eight metabolites differentially accumulated in the drought-tolerant genotype (X). Only L-leucine was significantly enriched in three pathways simultaneously, including valine, leucine and isoleucine biosynthesis, valine, leucine, and isoleucine degradation and glucosinolate biosynthesis (Figure 7C). The specific consistent upregulation of L-leucine may be an important mechanism for enhancing drought resistance in *E. sibiricus*.

### 2.6. Analysis of Metabolic Pathways in Drought Tolerance and Drought Sensitive Genotypes

**Figure 8:**
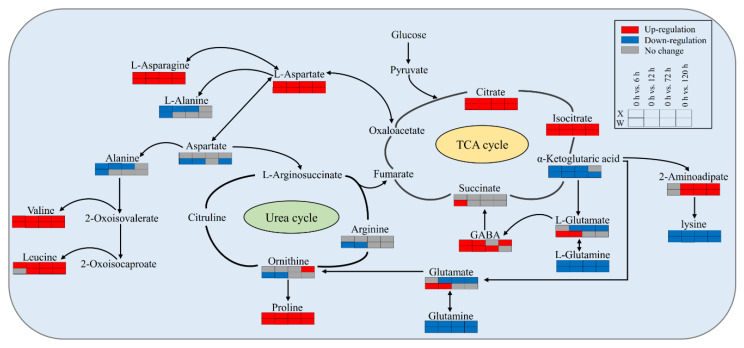
Analysis of the metabolic network in *E. sibiricus* under drought stress. The proposed metabolic pathways are based on the literature and KEGG database. Red indicates a significant increase, blue indicates a significant decrease, and gray indicates no significant change.

In order to comprehensively understand the changes in metabolites under drought stress, a metabolic pathway based on the literature and the KEGG database of metabolic pathways was proposed. The major known pathways including citrate cycle (TCA cycle), urea cycle, D-amino acid metabolism, alanine, aspartate, and glutamate metabolism, 2-oxocarboxylic acid metabolism, and biosynthesis of amino acids (Figure 8). Citric acid (TCA cycle), isocitrate (TCA cycle), l-aspartate (alanine, aspartate, and glutamate metabolism), l-asparagine (alanine, aspartate, and glutamate metabolism), valine (biosynthesis of amino acids), and proline (biosynthesis of amino acids) were significantly up-regulated at four treatment time points compared with 0 h. In contrast, lysine (2-oxocarboxylic acid metabolism), glutamine (biosynthesis of amino acids), and l-glutamine (alanine, aspartate, and glutamate metabolism) were significantly down-regulated compared with 0 h. The results indicated that drought stress can effectively stimulate the responses of these four metabolic pathways. 4-Aminobutanoate (GABA) was up-regulated during short-term (6 and 12 h) drought stress, and 2-aminoadipate was up-regulated during long-term drought stress in the drought-tolerant genotype (X) and drought-sensitive genotype (W). Interestingly, glutamate and l-glutamate showed the same cumulative pattern in the two genotypes, which showed down-regulated accumulation in the drought-tolerant genotype (X) under long-term stress and up-regulated accumulation in the drought-sensitive genotype (W) under long-term stress. This suggests that the difference in the cumulative pattern of glutamate and l-glutamate in the two genotypes *E. sibiricus* may be one of the main reasons for the difference in drought resistance.

## 3. Discussion

Understanding the drought-tolerance mechanisms and improving the drought tolerance of plants are critical for the food security of the growing global population [18]. Emerging studies have aimed to reveal the tolerance mechanisms of plants via biological responses, including phenological development [19], gene expression [20], morphological, and physiological responses [20]. On the other hand, the metabolic network should be reprogrammed to maintain the metabolic balance and to meet the demand for anti-stress agents [11]. In the present study, we measured the metabolite levels of drought-tolerant and drought-sensitive *E. sibiricus* accessions that treated with PEG using UPLC–MS and indicated that the drought response of *E. sibiricus* was a complex process involving various metabolites participating in the TCA cycle, starch and sucrose metabolism, flavonoids metabolism process, the urea cycle, D-amino acid metabolism, alanine, aspartate and glutamate metabolism, 2-oxocarboxylic acid metabolism, glucosinolate biosynthesis, and so on. We also found 27 key metabolite markers (Table S6) between sensitive and tolerant accessions, which could be considered as the references to evaluate the drought tolerance of *E. sibiricus* and related species.

Osmotic adjustment is an important process in maintaining cellular water potential, and osmotic regulation materials include carbohydrates, organic acids, free amino acids, and so on [21]. Many sugars are considered as the key metabolites and signal intermediates of plant biochemical pathways in response to drought stress [22]. Drought stress can promote the decomposition of storage sugars like starch into soluble sugars to regulate osmotic pressure and protect the biological structure of plants from stresses [23]. The decrease of starch content and the increased content of soluble sugars under drought stress were detected in maize and other plants [24,25,26]. In the current study, the contents of some soluble sugars in drought-tolerant *E. sibiricus* accessions, including turanose, melezitose, sucrose, melibiose, and so on, were much higher than those of drought-sensitive accessions under CK, which might provide the high osmotic potential and adaptive buffer of tolerance to response the stress. However, we did not observe that the soluble reducing sugar (maltose, glucose, fructose) content significantly increased with the extension of stress time, which could be attributed to the demand for energy metabolism at the later period of stress.

Another dominant metabolite in *E. sibiricus* is amino acids, whose accumulation is thought to help plants tolerate stress through osmoregulation, detoxification of ROS, and intracellular pH regulation [27]. Proline is a classical amino acid that accumulates in various plants in response to a wide range of abiotic stresses [28,29], especially as an important metabolite in response to drought [15,30]; in addition to being the substance for osmotic regulation, proline also stabilizes the subcellular structure and contributes to the detoxification of reactive oxygen species [31]. In this study, the cumulative amount of proline in the two genotypes of *E. sibiricus* during the whole drought stress process was significantly higher than that of CK. In addition, proline is a metabolite of glutamate, and a substantial supply of glutamate is needed when the rate of proline biosynthesis is increased [32]. Therefore, the significant decrease of glutamate accumulation detected in the drought-tolerant genotype (X) under long-term drought may be the result of the large amounts of proline required to cope with drought (Figure 8). This may be an important strategy for drought-tolerant genotypes to respond to drought. Citric acid, the key drought-tolerant metabolite of *E. sibiricus*, is an important intermediate product in the TCA cycle and can improve the tolerance of substances to abiotic stresses and the addition of citric acid to the exogenous source can improve the drought resistance of plants [33,34]. In this study, the cumulative amount of citric acid also increased significantly under drought pressure in the drought-tolerant genotype (X) and drought-sensitive genotype (W). Therefore, increasing endogenous citric acid levels through exogenous spraying may be an effective way to improve the drought resistance of *E. sibiricus*.

As the ‘specialized metabolites’ in plants, flavonoids are important active oxygen scavengers and nonenzymatic antioxidants that have multiple functions in plant development and response to biotic and abiotic stresses, and play an irreplaceable role in plant drought resistance [35,36]. The up-regulation of genes involved in flavonoids biosynthesis and the increased content of flavonoids under drought stress were observed in some species [37,38]. The relationship between its content and drought tolerance has been shown in studies on *Cajanus cajan*, in which, increased content increases drought resistance [39]. In this context, it seems that plants possessing a higher flavonoid content exhibited stronger drought resistance. However, the contents of almost all kinds of flavonoids showed no difference or were up-regulated in drought sensitive *E. sibiricus* accessions under CK and drought stresses when compared with the drought-tolerant genotype (Tables S2 and S4); the reason for these lines is the fact that stress sensitive accessions possess a lower “baseline”, which causes them to be exposed to a more severe stress [40].

Plants cultivated in the environment of high manual management will only experience interval short-term drought stress for a few hours to several days, which poses a challenge for plants to respond quickly and limits the damage caused by short-term drought stress [18]. However, for plants that naturally grow in the field, long-term drought stress might be experienced. Therefore, exploring the drought adaptation mechanisms of plants subjected to short- and long-term drought is equally important. In the current study, the metabolite levels under short-term and long-term drought stress in the two genotypes *E. sibiricus* were detected, and we found that the metabolite types were almost identical. What can be deduced is that the metabolic response under drought stress is a dynamic balance process, and energy metabolism might be more active in the later period of drought stress to survive.

When comparing the DMs of the drought-tolerant genotype (X) and drought-sensitive genotype (W) in the short-term response to drought, we found that some DMs of the two genotypes were completely different only in the short-term of drought, with only amino acid and its derivatives (L-glutamic acid, L-arginine, and L-ornithine) showing differences accumulating in the drought-sensitive genotype (W). Excepting the difference accumulation of amino acid and its derivatives (3-amino-2-methylpropanoic acid, Dl-glutamic acid), there were also carbohydrates and their derivatives (coniferin and galactinol), organic acid and its derivatives (R-aminobutyrate), flavones and flavonols (vitexin) and alkaloids and derivatives (trigonelline) differences accumulating in the drought-tolerant genotype (X). This suggests that the drought-tolerant genotype (X) exhibits a more complex metabolite accumulation process in the short-term response to drought stress. Drought stress is an extremely complex trait [41,42], in which many genes are involved in plant responses; accordingly, more metabolites are involved in responding to drought. Therefore, it is shown that drought-tolerant *E. sibiricus* responds to drought pressure by accumulating more kinds of metabolites under short-term drought stress, which was similar to the comparative proteomics study in *Triticum aestivum* L. [43]. Only under long-term drought stress, drought-tolerant genotype (X) and drought-sensitive genotype (W) exhibit the opposite pattern of metabolic accumulation as in the short-term drought, i.e., drought-tolerant genotype (X) accumulates fewer metabolites, and the drought-sensitive genotype (W) accumulates more. This could be because when a drought-tolerant genotype (X) is initially subjected to drought pressure, the signal of drought is transmitted faster and it quickly responds to drought pressure by modulating more gene expression [43,44,45]. With the prolongation of drought pressure time, the drought-tolerant genotype (X) has been able to cope with drought stress well through early gene expression and the stabilized level of metabolites [46]. In the later stage of drought, a drought-tolerant genotype (X) can reduce energy metabolism by reducing the transcriptional expression process and devote more energy to coping with drought stress [47]. However, the drought-sensitive genotype (W) is the opposite. Although less gene expression in the early stage can reduce energy consumption, when the drought time is prolonged, energy is not only needed to regulate gene expression, but also to cope with drought [48]. Therefore, we believe that the two genotypes of *E. sibiricus* show different coping patterns when subjected to drought stress, but it is obvious that the drought pressure response pattern of the drought-tolerant genotype (X) is more able to cope with long-term drought pressure.

**Figure 9:**
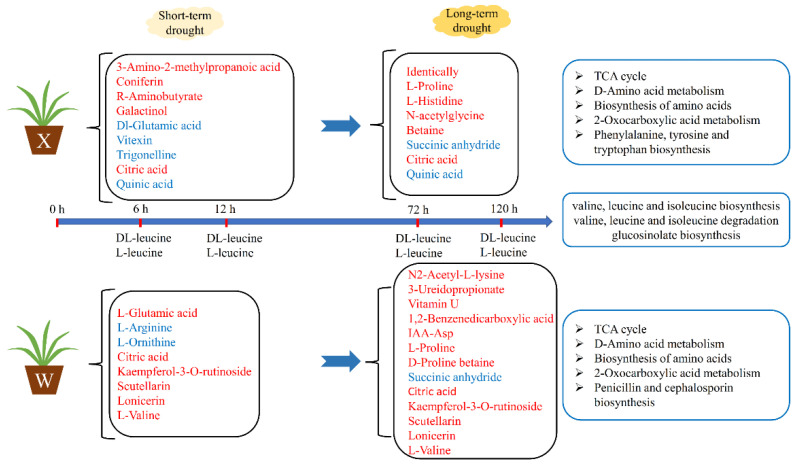
Patterns of response to drought stress in drought resistant and drought sensitive genotypes of *E. sibiricus*. The red font indicates increased, while the blue font indicates decreased.

Comparing the metabolites of the two genotypes in profile 19, it was found that there were eight unique metabolites in the drought-tolerant genotype (X), among which, the specific consistent up-regulation of DL-leucine and L-leucine may be another metabolic mode leading to the stronger drought resistance of the drought-tolerant genotype (X). Leucine was also found to be an important metabolite in pea (*Pisum sativum* L.) metabolism profiling, which showed a greater degree of accumulation in response to drought stress [49]. Therefore, leucine could be considered an important metabolic biomarker in response to drought stress in the drought-tolerant genotypes of *E. sibiricus*. Finally, based on the findings of our study, we have a simplified model of how drought-tolerant (X) and drought-sensitive (W) genotypes of *E. sibiricus* respond to short-term and long-term drought at the metabolic level (Figure 9).

## 4. Materials and Methods

### 4.1. Plant Materials

The seeds of 37 wild *E. sibiricus* accessions from major growing areas in China, including the QTP (Sichuan, Gansu, Tibet), Xinjiang, Inner Mongolia, Hebei Province were used. Full and disease−free seeds were selected and treated with 10% sodium hypochlorite solution (NaClO) for 15 min, then rinsed repeatedly with sterilized water. Next, the disinfected seeds were spread in 9-cm diameter petri dishes containing three sterile filter papers as the germination beds, then transplanted into pots when seedlings reached the three-leaf stage. The seedlings were grown in pots filled with clean river sand in a growth chamber at 25 °C with a 16-h light/8-h dark photoperiod. Hoagland nutrient solution was used for watering to maintain normal growth. When the seedlings grew to more than 5 tillers, they were soaked with half of Hoagland nutrient solution and 20% PEG-6000 for 5 days. Three independent biological replicates were set up for each wild *E. sibiricus*, and the drought-tolerant and drought-sensitive genotypes of *E. sibiricus* were screened by relative electrical conductivity (REC), relative water content (RWC) and phenotype scores (PS).

### 4.2. Drought Treatments of Two Genotype Materials and Sample Collection

The seed treatment and plant growth conditions of the two different genotypes were consistent with those of 37 wild *E. sibiricus*. Differently, the two drought-tolerant and drought-sensitive genotypes *E. sibiricus* were treated with half of Hoagland nutrient solution and 30% PEG-6000 for 0 (CK or X0 and W0), 6 (X6 and W6), 12 (X12 and W12), 72 (X72 and W72) and 120 (X120 and W120) h, in which, 6 and 12 h were short-term stress and 72 and 120 h were long-term stress. Samples were collected for independent biological replicates of the two genotypes at each time point, and a complete second leaf was collected from each tiller. A total of 30 samples were collected (three biological replicates × two genotypes × five time points). Fresh leaves were used to measure the REC and RWC of the two genotypes and leaves for metabolite analysis were snap-frozen in liquid nitrogen and stored at −80 °C until extraction.

### 4.3. RWC, REC and PS Measurement

Relative water content (RWC) was measured at all sampling timepoints. The fresh weight (FW) was immediately recorded after complete second leaf excision. The leaves were then soaked in distilled water for 12 h and the turgid weight (TW) was recorded. Finally, the dry weight (DW) was measured after the leaves were dried for 48 h at 65 °C. The RWC was calculated as RWC (%) = [(FW − DW)/(TW − DW)] × 100% [50]. All samples were prepared in three replicates. Student’s *t*-test was used to determine the statistical significance of each sample’s mean value.

Relative electrical conductivity (REC) measurements were determined by measuring electrolyte leakage from the leaves. Leaves of the same size were selected and immersed in a centrifuge tube with 10 mL deionized water for 6 h. After gentle shaking, the initial conductivity was measured and recorded as S1. Then, the centrifuge tube with leaves was placed in a boiling water bath and heated for 30 min. After cooling to normal temperature, the total conductivity was measured and recorded as S2. The REC was calculated as REC (%) = (S1/S2) × 100% [51].

Phenotypic scores (PS) were based on the method of Swain et al. [52], with minor modifications. The phenotypic score is a reference value that gives the plant the strength of drought resistance through the phenotypic difference of the plant under drought stress. The value of PS is mainly determined by the degree of leaf wilting, curling, and the number of yellowed leaves. The value of PS is 1–5 accurate to 0.1 from low to high (Figure S2).

### 4.4. Metabolomic Profiling

A widely targeted metabolome was used to obtain metabolomic profiling, which was performed in collaboration with Gene Denovo Biotechnology Co., Ltd. (Guangzhou, China), including mass spectrometry analyses and bioinformatics analysis. The method for widely targeted metabolic analysis mainly refers to that of Chen et al. [53], with appropriate modifications. In general, sample leaves were freeze-dried and ground for 60 s using a high-throughput tissue grinder at 45 Hz. An overnight extraction at 4 °C was performed with one hundred milligrams of lyophilized powder dissolved in 1.2 mL of 70% methanol solution (vortexed for 30 s every 30 s). Finally, the supernatant was collected after the extract was centrifuged at 12,000 rpm for 10 min, then filtered through a 0.22 µm pore size filter for ultra−performance liquid chromatography−tandem mass spectrometry (UPLC–MS/MS) analysis.

The sample extracts were analyzed using the UPLC–ESI–MS/MS system and the quality control samples (QC) were prepared by mixing all the sample extracts. In order to monitor the repeatability of the analysis process, QC was inserted every 5 samples. UPLC separation was performed using the same method as Yuan et al. [54]. Mass spectrometry analysis was performed by a triple quadrupole-linear ion trap mass spectrometer (Applied Biosystems 4500Q Trap UPLC/MS/MS system) equipped with an ESI Turbo Ion-Spray interface, operating according to previous studies. The Q1, Q3, RT (retention time), DP (declustering potential), and CE (collision energy) were used to the metabolite identification. In other words, the ionized material enters the triple quadrupole mass spectrometry system. Q1 quadrupole will screen the parent ions with a specific mass charge ratio (*m*/*z*). The parent ions screened enter Q2 crushing pool and then break into daughter ions with different mass charge ratios through certain Collision Energy (CE). These daughter ions were then entered into the Q3 quadrupole for further screening to obtain daughter ions with specific mass/charge ratio for subsequent detection [53]. Analyst 1.6.3 software (AB Sciex) was used for mass spectrometry data processing. Metabolites were qualitatively analyzed using the novogene database (Gene Denovo Biotechnology Co., Ltd., Guangzhou, China), MetWare database (Wuhan Meitehua Biotechnology Co., Ltd., Wuhan, China) and other public databases. The detection of the experimental samples using MRM (Multiple Reaction Monitoring) was based on house database. The Q3 were used to the metabolite quantification. The data files generated by UPLC-MS/MS were processed using the SCIEX OS Version 1.4 to integrate and correct the peak. The main parameters were set as follows: minimum peak height, 500; signal/noise ratio, 5; gaussian smooth width, 1. The area of each peak represents the relative content of the corresponding substance [53].

### 4.5. Data Analysis

Statistical analysis and histogram plotting of phenotypic data and metabolites were performed using Excel, and the significant differences in phenotypic data among samples were analyzed with IBM SPSS Statistics 27 software. The three phenotypic data were quantitatively transformed by the membership function method of fuzzy mathematics [55], and the average membership degree of each index was used as the comprehensive identification standard to compare the drought resistance of 37 wild *E. sibiricus*. RWC and PS were calculated using the Formula (1) and REC was calculated using Formula (2). The following formula is used to measure the indicator:

*U*(*X_i_*) = (*X_ij_* − *X_min i_*)/(*X_max I_* − *X_min i_*) (1)

*U*(*X_i_*) = 1 − (*X_ij_* − *X_min i_*)/(*X_max I_* − *X_min i_*) (2)

where *X_ij_* represents the *i*th determination index of the *j*th clone *U*(*X_i_*) ∈ [0, 1].

The Kyoto Encyclopedia of Genes and Genomes (KEGG) compound database was used to annotate the identified metabolites and the KEGG pathway database was used to map the annotated metabolites. KEGG pathway enrichment analysis was performed using the online data analysis platform OmicShare (https://www.omicshare.com/, accessed on 8 October 2022), and significance was determined by a hypergeometric test. In multivariate statistical analysis, high-dimensional and complex data can be simplified and original information retained more effectively. Principal component analysis (PCA) is a classic unsupervised pattern recognition multivariate statistical analysis method performed by the prcomp function in R. The data were unit variance scaled before PCA. The distance matrix of metabolite accumulation in all samples was calculated, and hierarchical clustering [56] was used to cluster all samples to form a dendrogram showing the similarity among samples. The degree of variation in metabolite composition and abundance between samples can be quantitatively analyzed through correlation data between samples. The closer the correlation is to 1, the higher the similarity of metabolic composition and abundance between samples. R was used for inter-sample Pearson’s correlation calculations and the pheatmap package [57] in R was used to draw heatmaps. Orthogonal partial least squares discriminant analysis (OPLS-DA) is a supervised pattern recognition method that was performed by R software using the R package MetabAnalystR [58]. Data were log-transformed (Log_2_) and centered on the mean before OPLS−DA. To avoid overfitting, permutation tests (200 permutations) were performed. Each comparison group has an independent OPLS-DA model, and 25 comparison groups have 25 OPLS-DA models. All the integral quantitative values of metabolites detected in each independent comparison group were used to perform the OPLS-DA. Variable importance for the projection (VIP ≥ 1) values and univariate statistical analysis *t*-test *p* value (*p* < 0.05) were extracted from the OPLS−DA results to identify differential metabolites (DMs) of other comparison groups. In addition, to explore the DMs between the two genotypes under drought stress, 15 samples from each genotype under 5 treatments were treated as a group, i.e., groups X and W. The comparison groups (X vs. W) were also subjected to an independent OPLS-DA. The DMs of the two genotypic comparison groups (X vs. W) were identified by S-plot (Figure S3) and the VIP values and t-test *p* value (*p* < 0.05) from the OPLS-DA. Figure S3 was the score and permutation test plots of OPLS-DA. In addition, DMs among each comparison group were identified by Venn diagram using the online data analysis platform OmicShare (https://www.omicshare.com/, accessed on 15 September 2022).

Weighted correlation network analysis (WGCNA) was performed with the online data analysis platform Omicsmart (https://www.omicsmart.com/home.html/, accessed on 3 October 2022) to identify key candidate metabolites associated with drought resistance in *E. sibiricus*. Pearson’s correlations between modules and traits were calculated and plotted with Omicsmart, to further screen out the key modules related to drought resistance in *E. sibiricus*. Trend analysis on the DMs of the two genotypes of *E. sibiricus* were performed by the online data analysis platform OmicShare (https://www.omicshare.com/, accessed on 16 October 2022), respectively, to explore the cumulative pattern of metabolites across different genotypes.

## 5. Conclusions

Drought stress altered the accumulation of metabolites and induced differential accumulation of metabolites in two different genotypes of *E. sibiricus*. In this study, amino acids and derivatives, flavonoids, flavones and flavonols, alcohols, and so on played an important role in the response to drought stress; Succinic anhydride was found to be a biomarker of prolonged drought stress in *E. sibiricus*, and the accumulation of succinic anhydride could determine whether *E. sibiricus* suffered from prolonged drought stress. A batch of drought stress markers and key metabolites in *E. sibiricus* were provided for the first time, which provided new resources for breeding *E. sibiricus* for drought resistance.

## Data Availability

The total ion chromatography (TIC) of the QC samples (Figure S4) were provided to verify the availability of the data.

## Supplementary Materials

The following supporting information can be downloaded at: https://www.mdpi.com/article/10.3390/ijms24010452/s1.
