# Supplementary material for: Comparative Metabolomic Studies of Siberian Wildrye (Elymus sibiricus L.): A New Look at the Mechanism of Plant Drought Resistance
Source: Int J Mol Sci. 2022 Dec 27;24(1):452. doi: 10.3390/ijms24010452 (PMC9820681; doi:10.3390/ijms24010452)
Supplement: Supplementary file 1 [file ijms-24-00452-s001.zip › ijms-2044401-supplementary figures.pdf]

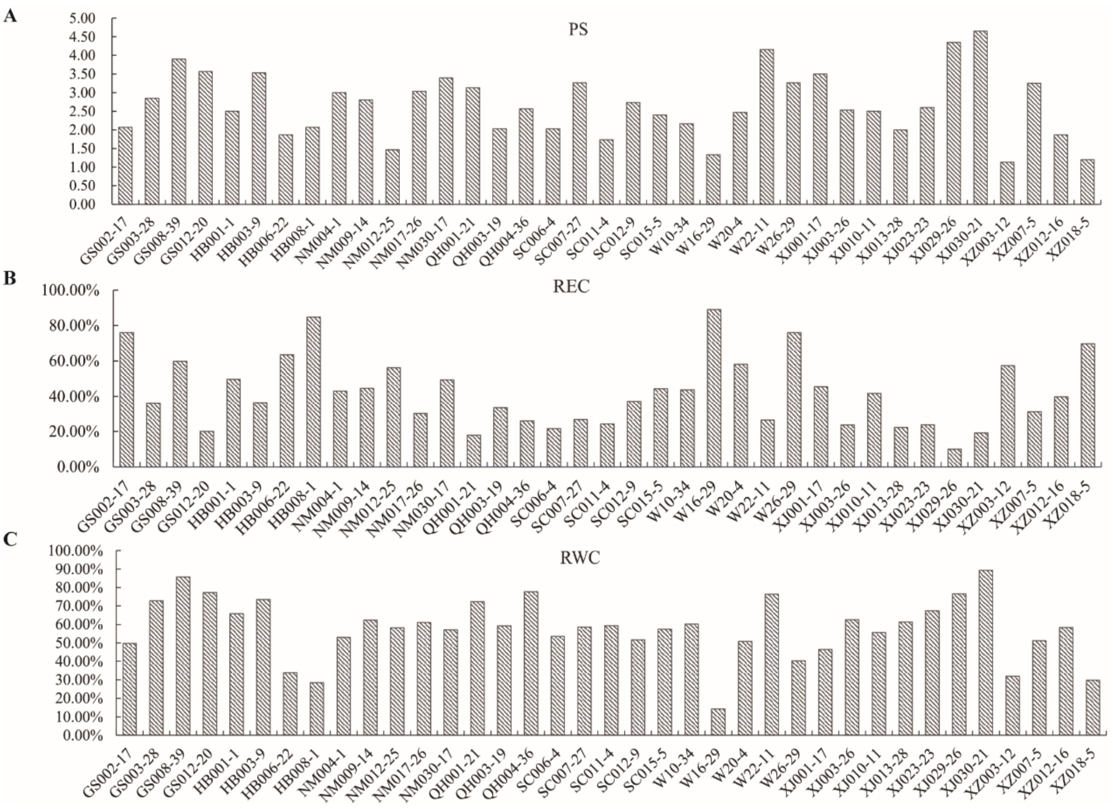

Figure S1. Phenotypic histogram of 37 wild *E. sibiricus* accessions. **A** shows the phenotypic score (PS), **B** shows the relative electrical conductivity (REC), and **C** shows the relative water content (RWC).

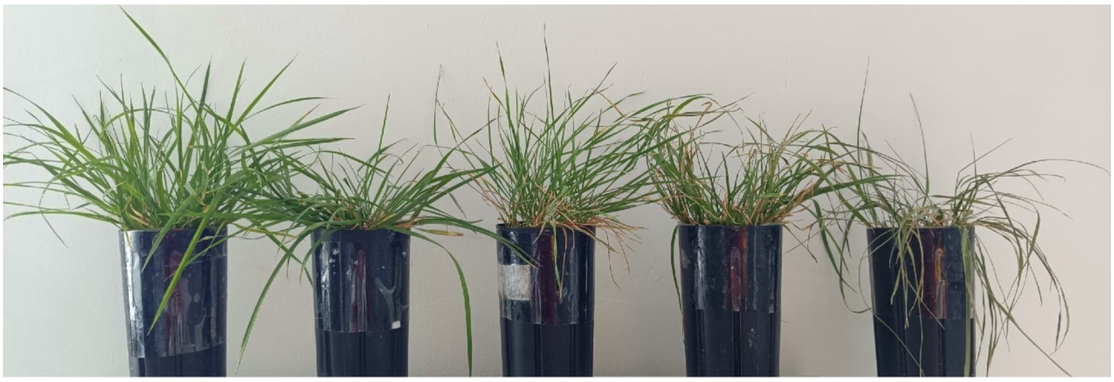

5 **4** **3** **2** **1**

Figure S2. The phenotypic scoring reference diagram of *E. sibiricus* shows **5**, **4**, **3**, **2**, and **1** points from left to right.

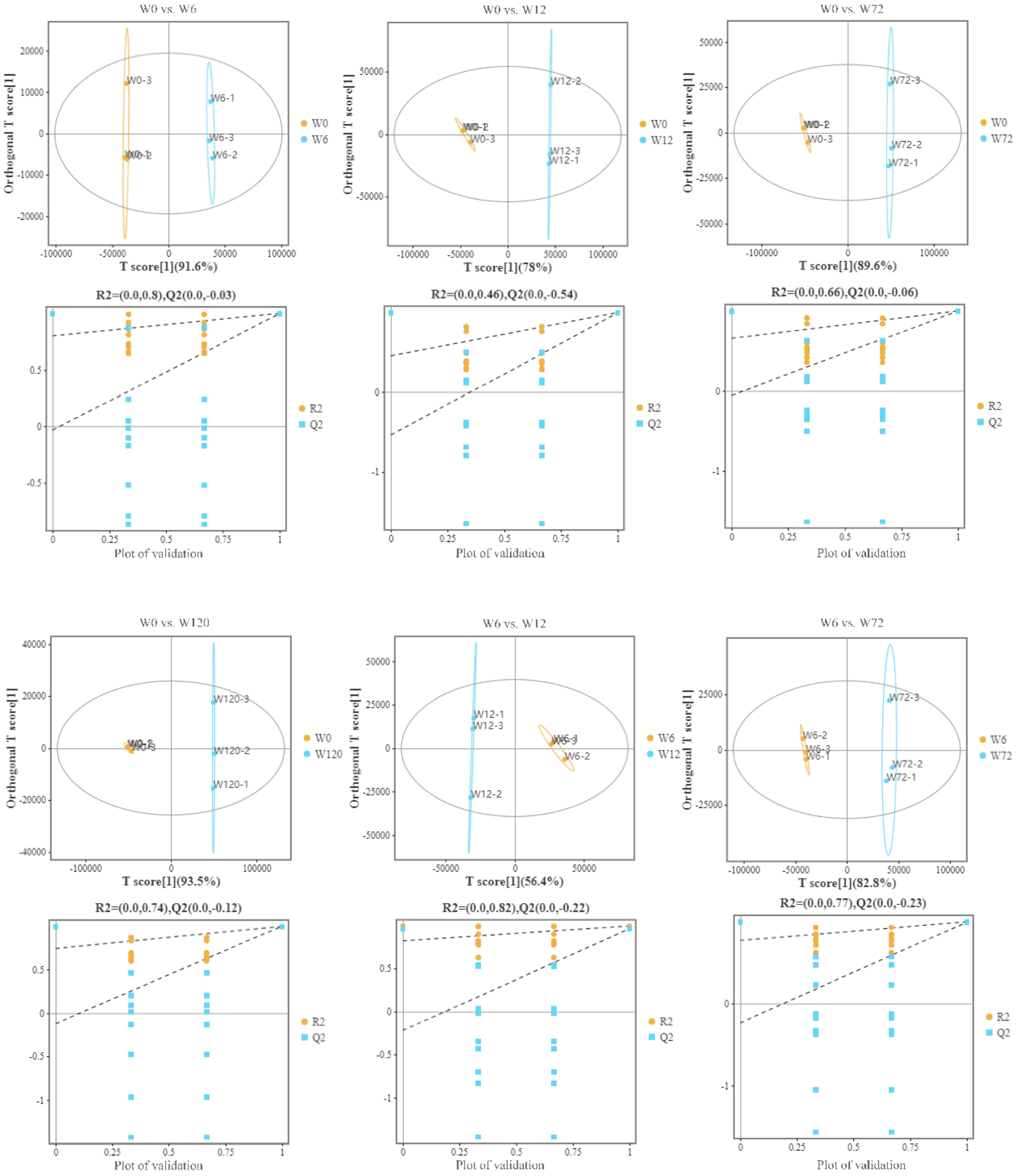
W0 vs. W6
W0 vs. W12
W0 vs. W72
W0 vs. W120
W6 vs. W12
W6 vs. W72
Orthogonal T score[1]
T score[1](91.6%)
T score[1](78%)
T score[1](89.6%)
T score[1](93.5%)
T score[1](56.4%)
T score[1](82.8%)
R2=(0.0,0.8),Q2(0.0,-0.03)
R2=(0.0,0.46),Q2(0.0,-0.54)
R2=(0.0,0.66),Q2(0.0,-0.06)
R2=(0.0,0.74),Q2(0.0,-0.12)
R2=(0.0,0.82),Q2(0.0,-0.22)
R2=(0.0,0.77),Q2(0.0,-0.23)
Plot of validation
W0
W6
W12
W72
W120
R2
Q2

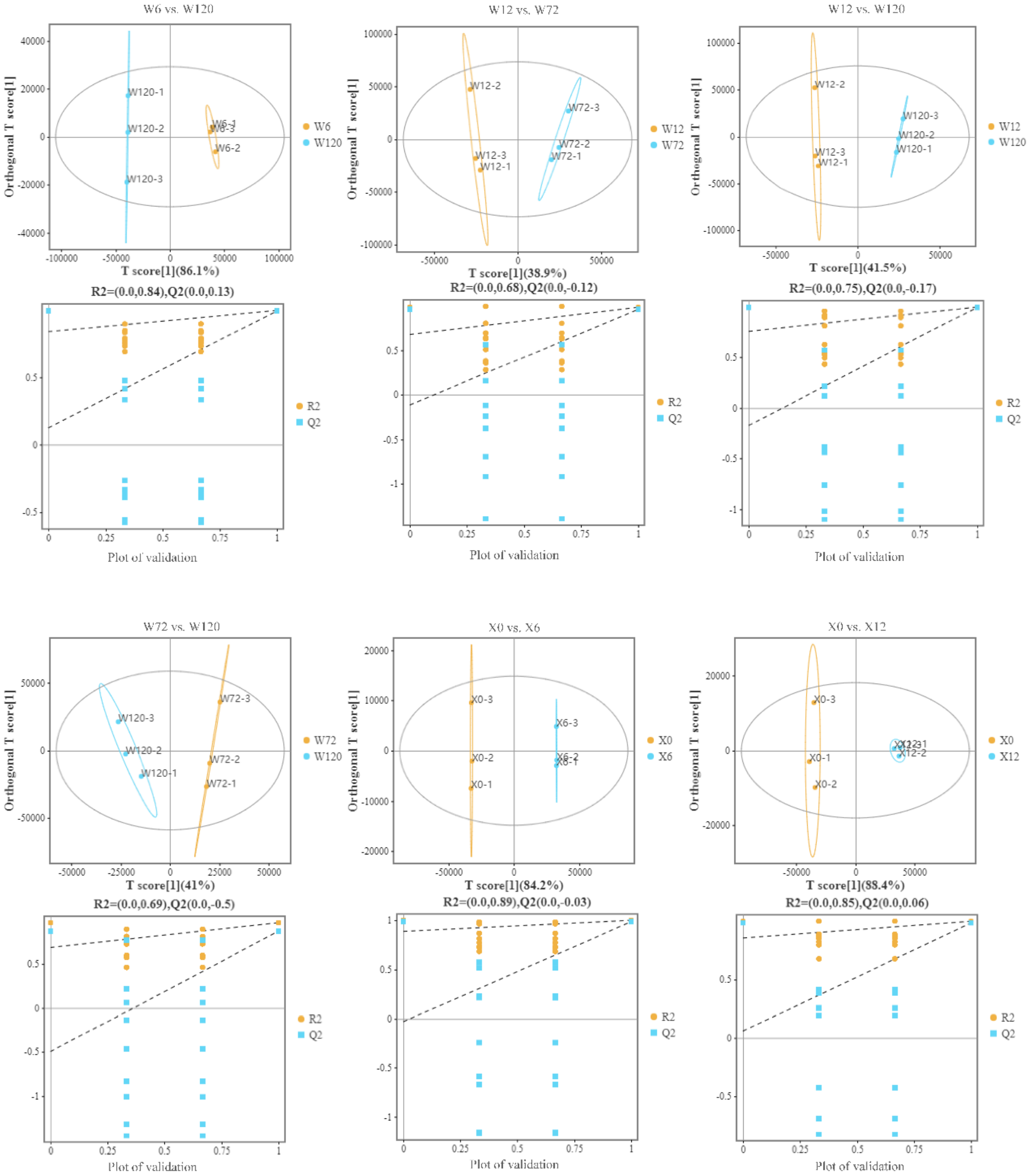
W6 vs. W120
Orthogonal T score[1]
T score[1](86.1%)
W6
W120
R2=(0.0,0.84),Q2(0.0,0.13)
Plot of validation
W12 vs. W72
T score[1](38.9%)
W12
W72
R2=(0.0,0.68),Q2(0.0,-0.12)
W12 vs. W120
T score[1](41.5%)
R2=(0.0,0.75),Q2(0.0,-0.17)
W72 vs. W120
T score[1](41%)
R2=(0.0,0.69),Q2(0.0,-0.5)
X0 vs. X6
T score[1](84.2%)
X0
X6
R2=(0.0,0.89),Q2(0.0,-0.03)
X0 vs. X12
T score[1](88.4%)
X12
R2=(0.0,0.85),Q2(0.0,0.06)
R2
Q2

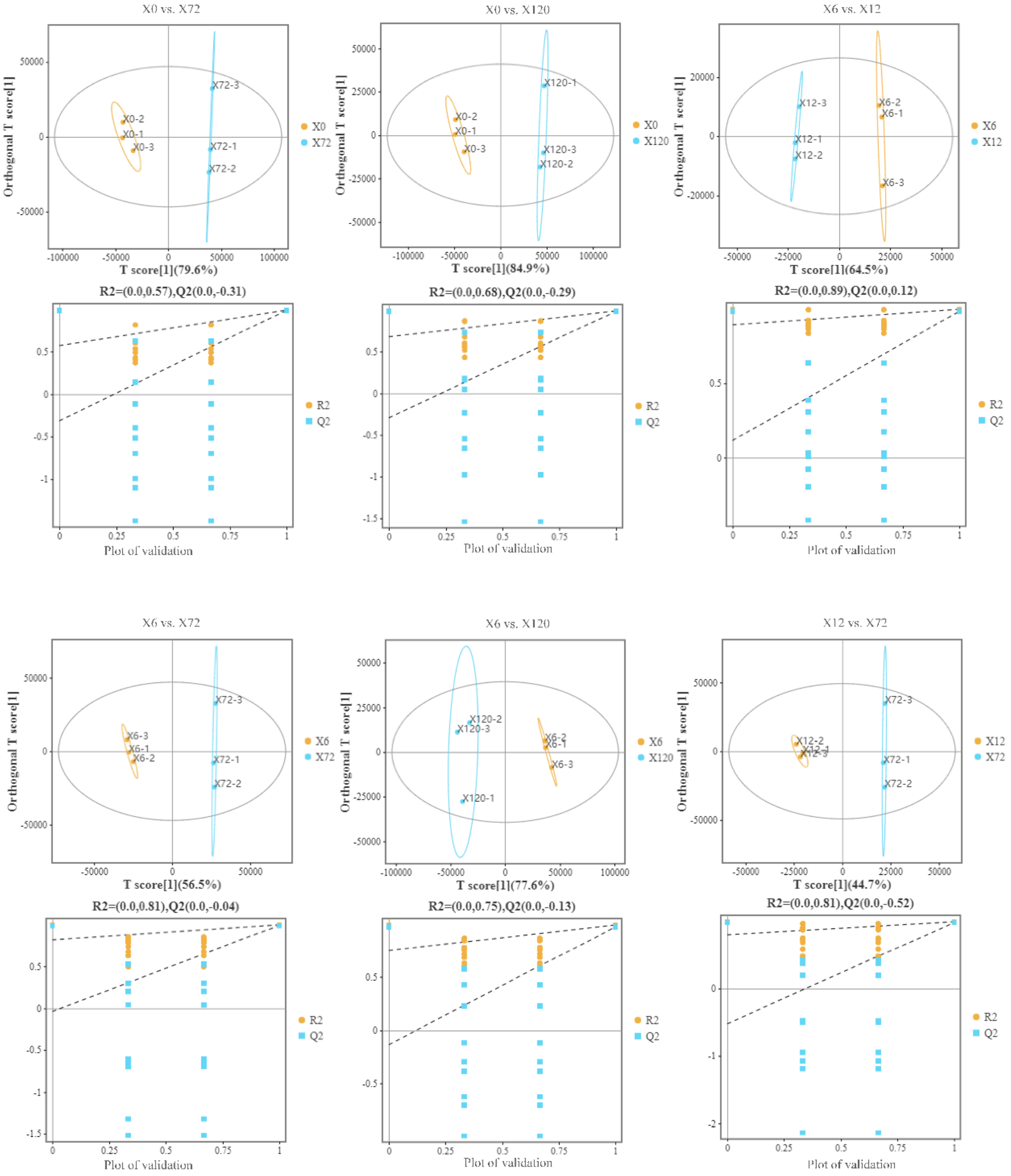
X0 vs. X72
Orthogonal T score[1]
T score[1](79.6%)
X0
X72
X0-1
X0-2
X0-3
X72-1
X72-2
X72-3
R2=(0.0,0.57),Q2(0.0,-0.31)
Plot of validation
R2
Q2
X0 vs. X120
Orthogonal T score[1]
T score[1](84.9%)
X0
X120
X0-1
X0-2
X0-3
X120-1
X120-2
X120-3
R2=(0.0,0.68),Q2(0.0,-0.29)
Plot of validation
R2
Q2
X6 vs. X12
Orthogonal T score[1]
T score[1](64.5%)
X6
X12
X6-1
X6-2
X6-3
X12-1
X12-2
X12-3
R2=(0.0,0.89),Q2(0.0,0.12)
Plot of validation
R2
Q2
X6 vs. X72
Orthogonal T score[1]
T score[1](56.5%)
X6
X72
X6-1
X6-2
X6-3
X72-1
X72-2
X72-3
R2=(0.0,0.81),Q2(0.0,-0.04)
Plot of validation
R2
Q2
X6 vs. X120
Orthogonal T score[1]
T score[1](77.6%)
X6
X120
X6-1
X6-2
X6-3
X120-1
X120-2
X120-3
R2=(0.0,0.75),Q2(0.0,-0.13)
Plot of validation
R2
Q2
X12 vs. X72
Orthogonal T score[1]
T score[1](44.7%)
X12
X72
X12-1
X12-2
X12-3
X72-1
X72-2
X72-3
R2=(0.0,0.81),Q2(0.0,-0.52)
Plot of validation
R2
Q2

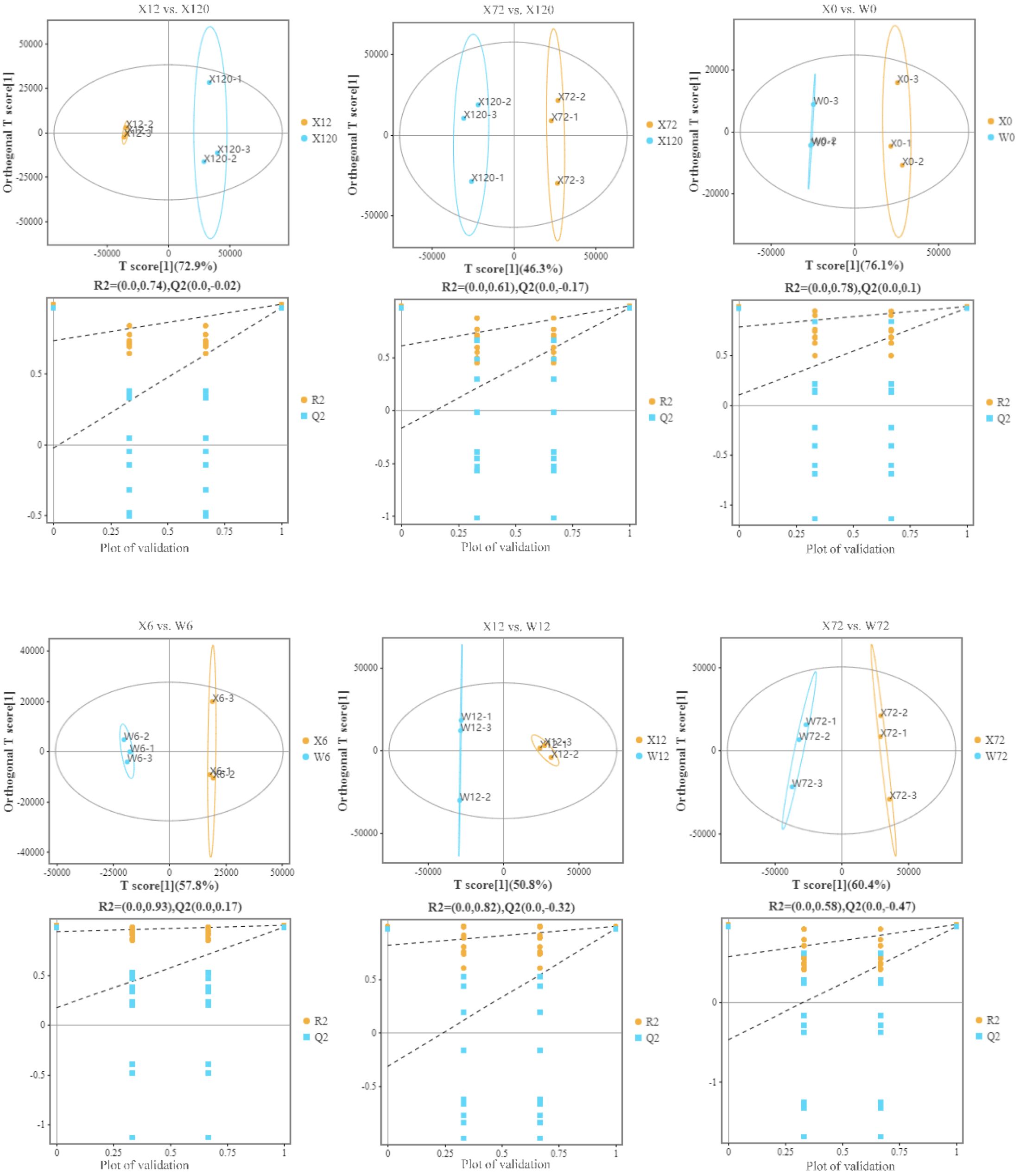
X12 vs. X120
Orthogonal T score[1]
T score[1](72.9%)
X12
X120
R2=(0.0,0.74),Q2(0.0,-0.02)
Plot of validation
R2
Q2
X72 vs. X120
Orthogonal T score[1]
T score[1](46.3%)
X72
X120
R2=(0.0,0.61),Q2(0.0,-0.17)
Plot of validation
R2
Q2
X0 vs. W0
Orthogonal T score[1]
T score[1](76.1%)
X0
W0
R2=(0.0,0.78),Q2(0.0,0.1)
Plot of validation
R2
Q2
X6 vs. W6
Orthogonal T score[1]
T score[1](57.8%)
X6
W6
R2=(0.0,0.93),Q2(0.0,0.17)
Plot of validation
R2
Q2
X12 vs. W12
Orthogonal T score[1]
T score[1](50.8%)
X12
W12
R2=(0.0,0.82),Q2(0.0,-0.32)
Plot of validation
R2
Q2
X72 vs. W72
Orthogonal T score[1]
T score[1](60.4%)
X72
W72
R2=(0.0,0.58),Q2(0.0,-0.47)
Plot of validation
R2
Q2

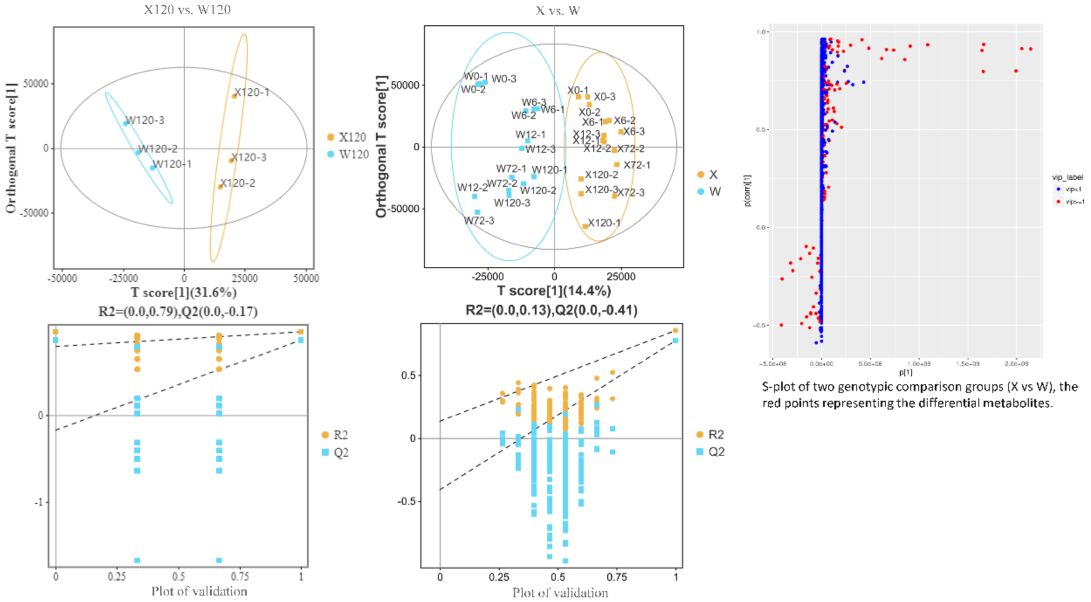

Figure S3. Validation and scoring plots of 25 comparison groups of OPLS-DA.

A

B

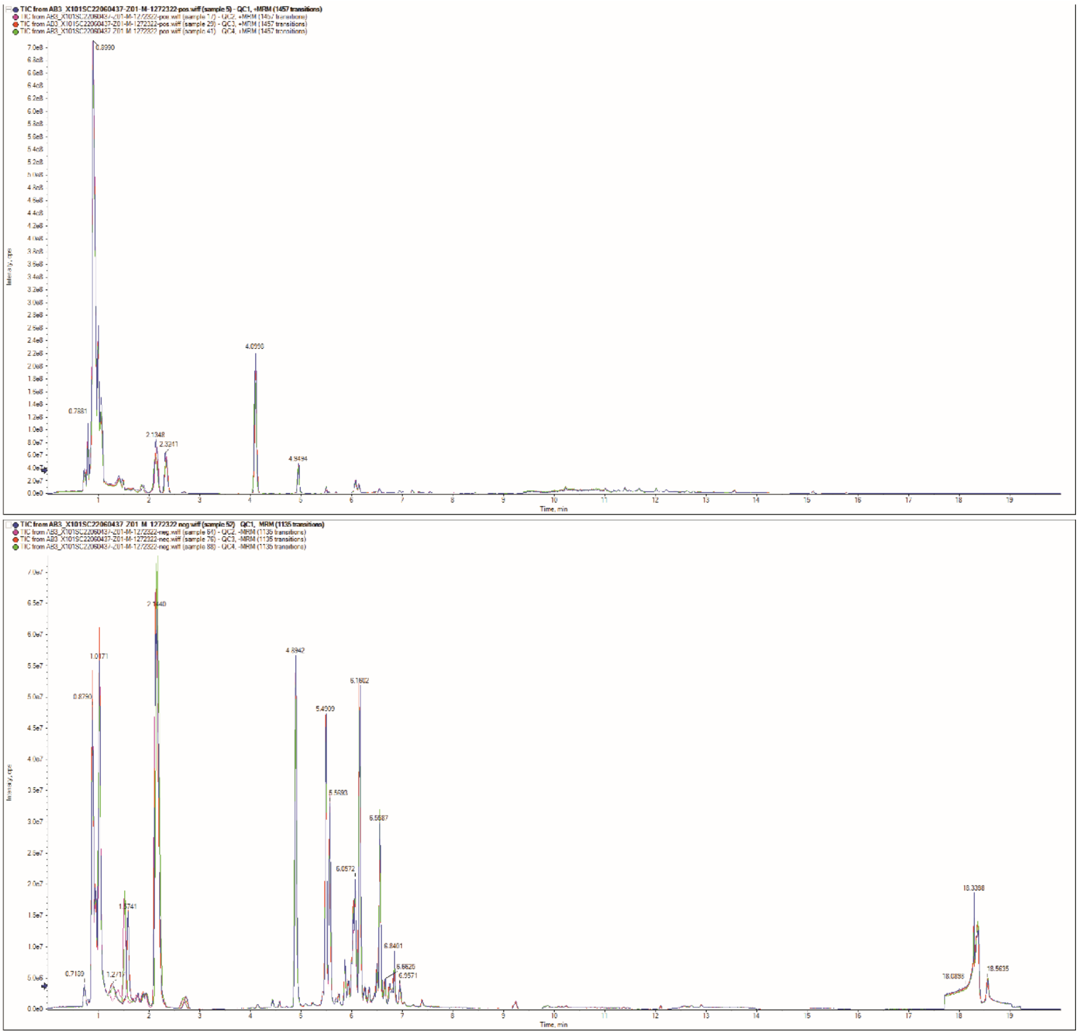

Figure S4. The total ion chromatography (TIC) of the QC samples; **A** stands for positive ion mode; **B** stands for negative ion mode.
