# Supplementary material for: Comparative Metabolomic Studies of Siberian Wildrye (Elymus sibiricus L.): A New Look at the Mechanism of Plant Drought Resistance
Source: Int J Mol Sci. 2022 Dec 27;24(1):452. doi: 10.3390/ijms24010452 (PMC9820681; doi:10.3390/ijms24010452)
Supplement: Supplementary file 1 [file ijms-24-00452-s001.zip › Table S1.pdf]

Table S1. Comprehensive evaluation and ranking of membership functions of 36 *Elymus sibiricus* phenotypic data.

**Sample ID**	**Membership function values of REC**	**Membership function values of PS**	**Membership function values of RWC**	**average**	**Order**	**Sample ID**	**Membership function values of REC**	**Membership function values of PS**	**Membership function values of RWC**	**Average**	**Order**
GS002-17	0.16	0.27	0.47	0.30	32	SC012-9	0.60	0.45	0.50	0.52	20
GS003-28	0.61	0.49	0.78	0.62	10	SC015-5	0.51	0.36	0.57	0.48	26
GS008-39	0.34	0.79	0.95	0.69	6	W10-34	0.52	0.29	0.61	0.48	27
GS012-20	0.79	0.69	0.84	0.77	4	W16-29	0.01	0.06	0.00	0.02	37
HB001-1	0.46	0.39	0.69	0.51	22	W20-4	0.36	0.38	0.49	0.41	29
HB003-9	0.60	0.68	0.79	0.69	7	W22-11	0.72	0.86	0.83	0.80	3
HB006-22	0.30	0.21	0.26	0.26	33	W26-29	0.16	0.61	0.35	0.37	30
HB008-1	0.06	0.27	0.19	0.17	35	XJ001-17	0.50	0.67	0.43	0.53	18
NM004-1	0.53	0.53	0.52	0.53	19	XJ003-26	0.75	0.40	0.64	0.60	13
NM009-14	0.51	0.47	0.64	0.54	17	XJ010-11	0.54	0.39	0.55	0.49	25
NM012-25	0.38	0.09	0.58	0.35	31	XJ013-28	0.76	0.25	0.63	0.55	16
NM017-26	0.67	0.54	0.62	0.61	12	XJ023-23	0.75	0.42	0.71	0.62	11
NM030-17	0.46	0.64	0.57	0.56	15	XJ029-26	0.90	0.91	0.83	0.88	2
QH001-21	0.81	0.57	0.77	0.72	5	XJ030-21	0.80	1.00	1.00	0.93	1
QH003-19	0.64	0.26	0.60	0.50	24	XZ003-12	0.37	0.00	0.24	0.20	34
QH004-36	0.72	0.41	0.84	0.66	8	XZ007-5	0.66	0.60	0.49	0.59	14
SC006-4	0.77	0.26	0.52	0.52	21	XZ012-16	0.57	0.21	0.59	0.45	28
SC007-27	0.71	0.61	0.59	0.64	9	XZ018-5	0.23	0.02	0.21	0.15	36
SC011-4	0.74	0.17	0.60	0.50	23						
