# Supplementary material for: Comparative Metabolomic Studies of Siberian Wildrye (Elymus sibiricus L.): A New Look at the Mechanism of Plant Drought Resistance
Source: Int J Mol Sci. 2022 Dec 27;24(1):452. doi: 10.3390/ijms24010452 (PMC9820681; doi:10.3390/ijms24010452)
Supplement: Supplementary file 1 [file ijms-24-00452-s001.zip › Table S6.pdf]

Table S6. Twenty-seven metabolites in response to drought in *E. sibiricus*.

index	Up/Down	Compounds	Class	Formula	Pathway
Com_1037_pos	Down	Lysine	Amino Acid And Derivatives	C6H14N2O2	-
Com_1093_pos	Up	L-Phenylalanine	Amino Acid And Derivatives	C9H11NO2	ko01100//Metabolic pathways;ko01110//Biosynthesis of secondary metabolites;ko01230//Biosynthesis of amino acids;ko02010//ABC transporters;ko00970//Aminoacyl-tRNA biosynthesis;ko01210//2-Oxocarboxylic acid metabolism;ko00940//Phenylpropanoid biosynthesis;ko00400//Phenylalanine, tyrosine and tryptophan biosynthesis;ko00960//Tropane, piperidine and pyridine alkaloid biosynthesis;ko00460//Cyanoamino acid metabolism;ko00360//Phenylalanine metabolism;ko00966//Glucosinolate biosynthesis
Com_202_neg	Up	Citric acid	Tca Cycle	C6H8O7	ko01100//Metabolic pathways;ko01110//Biosynthesis of secondary metabolites;ko01120//Microbial metabolism in diverse environments;ko01230//Biosynthesis of amino acids;ko01200//Carbon metabolism;ko01210//2-Oxocarboxylic acid metabolism;ko00630//Glyoxylate and dicarboxylate metabolism;ko00250//Alanine, aspartate and glutamate metabolism;ko00020//Citrate cycle (TCA cycle)
Com_327_neg	Down	2,2-Dimethylsuccinic acid	Organic Acid And Its Derivatives	C6H10O4	-

Com_363_neg	Down	L-Glutamine	Amino Acid And Derivatives	C5H10N2O3	ko01100//Metabolic pathways;ko01120//Microbial metabolism in diverse environments;ko01230//Biosynthesis of amino acids;ko02010//ABC transporters;ko00230//Purine metabolism;ko00240//Pyrimidine metabolism;ko00970//Aminoacyl-tRNA biosynthesis;ko00630//Glyoxylate and dicarboxylate metabolism;ko00250//Alanine, aspartate and glutamate metabolism;ko00220//Arginine biosynthesis;ko00750//Vitamin B6 metabolism;ko00910//Nitrogen metabolism
Com_523_pos	Down	Ala-gly	Amino Acid And Derivatives	C5H10N2O3	-
Com_552_pos	Up	N-Methyl-a-aminoisobutyric acid	Amino Acid And Derivatives	C5H11NO2	-
Com_572_pos	Up	7-methylguanine	Purines and purine derivatives	C6H7N5O	-
Com_580_pos	Down	Cycloleucine	Amino Acid And Derivatives	C6H11NO2	-
Com_620_pos	Down	Dl-Alanyl-glycine	Amino Acid And Derivatives	C5H10N2O3	-
Com_621_pos	Up	Dl-Indole-3-lactic acid	Indoles and derivatives	C11H11NO3	-
Com_624_pos	Up	Proline	Amino Acid And Derivatives	C5H9NO2	-
Com_625_pos	Up	Valine	Amino Acid And Derivatives	C5H11NO2	-

Com_626_pos	Down	Glutamine	Amino Acid And Derivatives	C5H10N2O3	-
Com_637_pos	Up	L-Tryptophan	Amino Acid And Derivatives	C11H12N2O2	ko01100//Metabolic pathways;ko01110//Biosynthesis of secondary metabolites;ko01230//Biosynthesis of amino acids;ko00970//Aminoacyl-tRNA biosynthesis;ko01210//2-Oxocarboxylic acid metabolism;ko00260//Glycine, serine and threonine metabolism;ko00380//Tryptophan metabolism;ko00400//Phenylalanine, tyrosine and tryptophan biosynthesis;ko00966//Glucosinolate biosynthesis;ko00901//Indole alkaloid biosynthesis
Com_670_pos	Down	DL-Citrulline	Amino Acid And Derivatives	C6H13N3O3	-
Com_673_pos	Up	D-Proline	Amino Acid And Derivatives	C5H9NO2	ko01100//Metabolic pathways;ko00330//Arginine and proline metabolism
Com_682_pos	Up	HBOA	Organoheterocyclic compounds	C8H7NO3	ko01110//Biosynthesis of secondary metabolites;ko00402//Benzoxazinoid biosynthesis
Com_728_pos	Up	isoleucine	Amino Acid And Derivatives	C6H13NO2	-
Com_729_pos	Down	D-glutamine	Amino Acid And Derivatives	C5H10N2O3	ko01100//Metabolic pathways
Com_775_pos	Up	D-Phenylalanine	Amino Acid And Derivatives	C9H11NO2	ko01100//Metabolic pathways;ko00360//Phenylalanine metabolism
Com_778_pos	Up	DL-Norvaline	Amino Acid And Derivatives	C5H11NO2	-
Com_863_pos	Up	trans-3-Hydroxy-L-proline	Amino Acid And Derivatives	C5H9NO3	ko01100//Metabolic pathways;ko00330//Arginine and proline metabolism

Com_888_pos	Up	6-Aminocaproic acid	Fatty Acyls	C6H13NO2	ko01100//Metabolic pathways;ko01120//Microbial metabolism in diverse environments
Com_898_pos	Down	DL-Glutamine	Amino Acid And Derivatives	C5H10N2O3	-
Com_900_pos	Down	L-lysine	Amino Acid And Derivatives	C6H14N2O2	ko01100//Metabolic pathways;ko01110//Biosynthesis of secondary metabolites;ko01120//Microbial metabolism in diverse environments;ko01230//Biosynthesis of amino acids;ko02010//ABC transporters;ko00970//Aminoacyl-tRNA biosynthesis;ko01210//2-Oxocarboxylic acid metabolism;ko00310//Lysine degradation;ko00960//Tropane, piperidine and pyridine alkaloid biosynthesis;ko00300//Lysine biosynthesis;ko00780//Biotin metabolism
Com_96_neg	Up	Isocitrate	Organic Acid And Its Derivatives	C6H8O7	ko01100//Metabolic pathways;ko01110//Biosynthesis of secondary metabolites;ko01120//Microbial metabolism in diverse environments;ko01230//Biosynthesis of amino acids;ko01200//Carbon metabolism;ko01210//2-Oxocarboxylic acid metabolism;ko00630//Glyoxylate and dicarboxylate metabolism;ko00020//Citrate cycle (TCA cycle)
